# N‐terminal pro‐B‐type natriuretic peptide for prediction of ventricular arrhythmias: Data from the SMASH study

**DOI:** 10.1002/clc.24074

**Published:** 2023-07-03

**Authors:** N. Sourour, E. Riveland, P. Næsgaard, H. Kjekshus, A. I. Larsen, T. Omland, H. Røsjø, P. L. Myhre

**Affiliations:** ^1^ Department of Cardiology, Division of Medicine Akershus University Hospital Lørenskog Norway; ^2^ K.G. Jebsen Center for Cardiac Biomarkers, Institute of Clinical Medicine Oslo Norway; ^3^ Department of Cardiology Stavanger University Hospital Stavanger Norway; ^4^ Institute of Clinical Sciences University of Bergen Bergen Norway; ^5^ Division for Research and Innovation Akershus University Hospital Lørenskog Norway

**Keywords:** ICD, implantable cardioverter defibrillator, N‐terminal pro‐B‐type natriuretic peptide, NT‐proBNP, sudden cardiac death, ventricular arrhythmias

## Abstract

**Background:**

Elevated N‐terminal pro‐B‐type natriuretic peptide (NT‐proBNP) concentrations predict heart failure (HF) and mortality, but whether NT‐proBNP predicts ventricular arrhythmias (VA) is not clear.

**Hypothesis:**

We hypothesize that high NT‐proBNP concentrations associate with the risk of incident VA, defined as adjudicated ventricular fibrillation or sustained ventricular tachycardia.

**Methods:**

In a prospective, observational study of patients treated with implantable cardioverter defibrillator (ICD), we analyzed NT‐proBNP concentrations at baseline and after mean 1.4 years in association to incident VA.

**Results:**

We included 490 patients (age 66 ± 12 years, 83% men) out of whom 51% had a primary prevention ICD indication. The median NT‐proBNP concentration was 567 (25–75 percentile 203–1480) ng/L and patients with higher concentrations were older with more HF and ICD for primary prevention. During mean 3.1 ± 0.7 years, 137 patients (28%) had ≥1 VA. Baseline NT‐proBNP concentrations were associated with the risk of incident VA (hazard ratio [HR]: 1.39, 95% confidence interval [95% CI]: 1.22–1.58, *p* < .001), HF hospitalizations (HR: 3.11, 95% CI: 2.53–3.82, *p* < .001), and all‐cause mortality (HR: 2.49, 95% CI: 2.04–3.03, *p* < .001), which persisted after adjusting for age, sex, body mass index, coronary artery disease, HF, renal function, and left ventricular ejection fraction. The association with VA was stronger in secondary versus primary prevention ICD indication: HR: 1.59 (95% CI: 1.34–1.88 C‐statistics 0.71) versus HR: 1.24, 95% CI: 1.02–1.51, C‐statistics 0.55), *p*‐for‐interaction = 0.06. Changes in NT‐proBNP during the first 1.4 years did not associate with subsequent VA.

**Conclusions:**

NT‐proBNP concentrations are associated with the risk of incident VA after adjustment for established risk factors, with the strongest association in patients with a secondary prevention ICD indication.

## Graphical abstract

NT‐proBNP concentrations are associated with the risk of incident ventricular arrhythmias in patients treated with implantable cardioverter defibrillator (ICD), with a stronger association among patients treated with ICD for secondary versus primary prevention. Higher NT‐proBNP levels were also associated with the risk of heart failure hospitalization and all‐cause mortality.

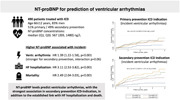

## 1 INTRODUCTION

Ventricular arrhythmia (VA) is an important cause of sudden cardiac death (SCD) globally.^1^, ^2^ Prediction of risk for VA and patients selection for treatment with implantable cardioverter defibrillator (ICD) are challenging due to a large number of heart disease conditions that can result in VA and subsequently SCD.

N‐terminal pro‐B‐type natriuretic peptide (NT‐proBNP) is a natriuretic peptide secreted by ventricular cardiomyocytes in response to cardiac stress mainly due to congestive heart failure (HF).^3^ Elevated levels of NT‐proBNP are predictive of poor prognosis in patients with HF, asymptomatic left ventricular (LV) dysfunction and coronary artery disease (CAD).^4^, ^5^ Previous studies have demonstrated that higher NT‐proBNP concentrations are associated with increased risk of SCD in patients with chronic HF, ischemic heart disease, hypertrophic cardiomyopathy, and in the general population.^6^, ^7^, ^8^, ^9^ Most studies have analyzed NT‐proBNP in association with clinically suspected SCD^6^, ^8^, ^10^ and few have investigated the association with recordings of VA,^7^, ^11^ which is an outcome measure more relevant for risk stratification and patients selection for ICD treatment. HF with reduced LV ejection fraction (LVEF) is the most frequent primary prevention ICD indication, and these patients therefore typically have higher NT‐proBNP concentrations than patients with a secondary prevention ICD indication.

The primary aim of this study was to assess the association between NT‐proBNP and device‐recorded and adjudicated incident VA. The secondary aim was to assess the association between NT‐proBNP and the risk of HF hospitalization and death.

## 2 METHODS

### 2.1 Study design and study population

SMASH (Scandinavian Multicenter study to Advance risk Stratification in Heart disease – ventricular arrhythmia) 1 is a prospective, observational, multicenter study.^12^ Patients treated with ICD who were ≥18 years old with life expectancy >2 years were screened for inclusion (inclusion criteria are summarized in Supporting Information: Figure 1). Study participants were included during regular outpatient visits at the Departments of Cardiology at Akershus University Hospital and Stavanger University Hospital between August 2016 and March 2018. All patients were invited to a follow‐up visit between 1 and 2 years after inclusion.

Patients underwent a physical examination at the baseline and follow‐up visit including measurement of blood pressure (average of the second and third measurements) and heart rate after 5 min rest. Body weight and height were measured, and body mass index (BMI) was calculated. Information regarding previous medical history and New York Heart Association (NYHA) functional class was obtained from a structured interview and by a thorough review of the electronic health records. Glomerular filtration rate (eGFR) was estimated from creatinine measured in routine blood samples. The most recent measurement of LVEF by echocardiography or cardiac magnetic imaging was recorded. CAD was defined as established chronic coronary syndrome or previously experienced acute coronary syndromes.

### 2.2 Analysis of NT‐proBNP

At both visits, patients donated blood specimens by venipuncture, performed by trained study nurses. Samples for the study biobank were temporarily stored at 4°C, centrifuged at 2000*g* for 10 min and then transferred into aliquots that were frozen and stored at −80°C at Akershus University Hospital. Serum samples that had not previously been thawed were used to measure NT‐proBNP, which was analyzed by the electrochemiluminescence immunoassay Elecsys on the Cobas e 801 platform (Roche Diagnostics). The coefficients of variations reported by the manufacturer were 2.5% at 127 ng/L and 1.3% at 1706 ng/L.

### 2.3 Outcome measures

The primary outcome in the SMASH study was incident VA, defined as episodes of ventricular tachycardia (VT) or ventricular fibrillation (VF) resulting in appropriately delivered ICD therapies, that is, electrical shock or antitachycardia pacing, or sustained ventricular tachyarrhythmia (>100 b.p.m. and >30 s). Events with VA were obtained from ICD recordings and adjudicated by experienced cardiac electrophysiologists that were blinded to study biomarker concentrations. Study investigators also reviewed the ICD recordings and the reports in the electronic healthcare record and validated real events from artifacts and ensured that appropriate therapies were separated from inappropriate ICD therapies. Only events validated as real VAs were included as outcomes in the study. HF hospitalization and death from any cause were secondary endpoints, registered by review of the electronic healthcare records of the patients, with linkage to the National Death Registry.

### 2.4 Statistical analysis

Values are reported as *N* (%) and median (Quartile 1 to Quartile 3) for skewed and mean ± SD for normally distributed variables. NT‐proBNP had a non‐normal distribution according to the Shapiro–Wilk normality test, and log‐transformed values were therefore used in all regression analyses. Categorical and continuous variables were compared using the *χ* ^2^ test for binary variables, analysis of variance for parametric continuous variables, and the Kruskal–Wallis test for nonparametric continuous variables. Baseline characteristics were compared for trend across quartiles of baseline NT‐proBNP using linear and logistic regression models. Independent predictors of higher baseline NT‐proBNP concentrations were determined using multivariable linear regression analysis. The associations between baseline concentrations of NT‐proBNP and time to first event for each of the endpoints (incident VA, HF hospitalization, and death in separate analyses) were examined in unadjusted and adjusted Cox proportional hazard regression models. Multivariable Model 1 was adjusted for age, sex, and BMI, and Model 2 was additionally adjusted for CAD, HF, eGFR, and LVEF. Harrell's C‐statistics was calculated to assess the performance of NT‐proBNP to discriminate between patients based on time to event. We performed interaction analysis to determine whether the association between NT‐proBNP and VA was different in patients with primary versus secondary prevention ICD indication. We used Kaplan–Meier plots to visualize the proportion of patients with endpoint events over time by quartiles of baseline NT‐proBNP.

In patients with available NT‐proBNP concentrations at the follow‐up visit, we used Wilcoxon signed‐rank test to analyze changes from the baseline samples. Relative changes in NT‐proBNP from baseline to follow‐up was calculated by dividing the follow‐up concentration with the baseline concentration. This ratio was log‐transformed and analyzed in landmark Cox regression models for events after the date of the follow‐up. All statistical analyses were performed using Stata Software (version 17, Stata Corp.). A two‐sided *p* < .05 was considered statistically significant.

## 3 RESULTS

### 3.1 Baseline characteristics

In the SMASH 1 Study, we included 495 patients treated with ICD, one withdrew from the study and among the remaining patients 490 (99%) had available study blood samples and were included in this analysis (Supporting Information: Figure 1). The mean age was 66 ± 12 years and 83% were men with a mean BMI of 28 ± 5 kg/m^2^ and LVEF of 40 ± 13%. Most patients had comorbid conditions, including CAD (64%), previous acute myocardial infarction (AMI, 57%) and HF (80%). The time from ICD implantation to study inclusion was 5.1 ± 6.6 years. Two‐hundred and fifty (51%) patients had a primary prevention ICD indication and 135 (28%) patients had cardiac resynchronization therapy with ICD indication. Baseline medications included 458 (94%) on β‐blockers, 395 (81%) on renin–angiotensin system inhibitors, and 84 (17%) on antiarrhythmic drugs. Among patients with ICD for primary prevention, HF was the indication in 225 patients (90%; mean LVEF 35 ± 11%), whereas 25 patients (10%; mean LVEF 57 ± 6%) had a non‐HF indication, predominantly cardiomyopathy (*n* = 17) (Supporting Information: Table 1).

### 3.2 Predictors of higher NT‐proBNP concentrations

The median (Q1–Q3) concentration of NT‐proBNP in the total population was 567 (203–1480) ng/L. Patients with higher NT‐proBNP concentrations were older, had lower BMI, lower LVEF, and higher NYHA functional class (Table 1). Patients with high NT‐proBNP concentrations were also more likely to have a greater burden of comorbidities, including HF, diabetes, CAD, previous AMI, and worse renal function. In multivariable regression models, older age, lower BMI, history of HF, absence of cardiomyopathy, NYHA class III–IV, lower LVEF, and lower eGFR levels were independent predictors of higher NT‐proBNP concentrations (Supporting Information: Table 2).

**Table 1:** Baseline characteristics of patients according to baseline NT‐proBNP quartiles.

	NT‐proBNP Q1 *n* = 123	NT‐proBNP Q2 *n* = 122	NT‐proBNP Q3 *n* = 123	NT‐proBNP Q4 *n* = 122	*p* for trend
NT‐proBNP range, ng/L	16–203	207–567	568–1480	1488–35 000	
Age, years	57.9 ± 12.6	67.3 ± 9.9	68.3 ± 9.8	71.0 ± 12.8	<.001
Male sex	98 (79.7%)	107 (87.7%)	96 (78.0%)	106 (87.6%)	.35
BMI, kg/m^2^	28.3 ± 5.0	28.9 ± 4.1	27.9 ± 4.9	25.7 ± 4.2	<.001
Systolic blood pressure, mmHg	125 ± 17	128 ± 20	125 ± 21	122 ± 23	.11
Diabetes mellitus	12 (9.8%)	23 (18.9%)	25 (20.3%)	34 (27.9%)	<.001
CAD	53 (43.8%)	86 (70.5%)	82 (68.3%)	88 (73.3%)	<.001
Previous AMI	40 (32.8%)	81 (66.4%)	74 (60.7%)	81 (66.9%)	<.001
HF	63 (51.2%)	102 (83.6%)	112 (91.1%)	114 (95.1%)	<.001
LVEF, %	50 ± 11	42 ± 11	36 ± 11	33 ± 12	<.001
NYHA Class III–IV	2 (1.6%)	16 (13.1%)	14 (11.4%)	20 (16.4%)	<.001
Cardiomyopathy	16 (13.0%)	5 (4.1%)	5 (4.1%)	8 (6.6%)	.06
Previous documentation of VA	66 (54.1%)	85 (69.7%)	68 (55.7%)	63 (51.6%)	.30
Primary ICD indication	57 (46.3%)	48 (39.3%)	66 (53.7%)	77 (63.6%)	<.001
Estimated GFR, ml/min/1.73m^2^	86 ± 21	78 ± 22	72 ± 23	58 ± 22	<.001
Baseline medications					
β‐blockers	106 (86.2%)	116 (95.1%)	119 (96.7%)	117 (95.9%)	.002
Angiotensin‐converting enzyme inhibitors	53 (43.1%)	61 (50.4%)	72 (58.5%)	60 (49.2%)	.19
Angiotensin II receptor blockers	28 (22.8%)	43 (35.2%)	34 (27.6%)	44 (36.1%)	.08
Mineralocorticoid receptor antagonists	21 (17.1%)	46 (37.7%)	59 (48.0%)	55 (45.1%)	<.001
Antiarrhythmic drugs	10 (8.1%)	18 (14.8%)	28 (22.8%)	28 (23.0%)	<.001

Abbreviations: AMI, acute myocardial infarction; BMI, body mass index; CAD, coronary artery disease; GFR, glomerular filtration rate; HF, heart failure; ICD, implantable cardioverter defibrillator; LVEF, Left ventricular ejection fraction; NT‐proBNO, N‐terminal pro‐B‐type natriuretic peptide; NYHA, New York Heart Association; VA, ventricular arrhythmia.

### 3.3 NT‐proBNP in association with incident VAs

**Figure 1:**
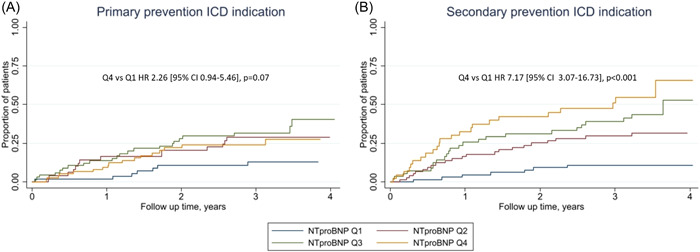
Association between baseline concentrations of N‐terminal‐pro B‐type natriuretic peptide (NT‐proBNP) and time to ventricular arrhythmia in patients with (A) primary prevention implantable cardioverter defibrillator (ICD) indication and (B) secondary prevention ICD indication. Stratified by quartiles of NT‐proBNP and *p* is for Quartile 4 versus Quartile 1.

During a mean follow‐up of 3.10 ± 0.74 years, 137 (28%) patients experienced at least one episode of VA, among whom 126 had VT, 47 had VF, and 120 had appropriate ICD therapy. Higher NT‐proBNP concentrations were associated with greater risk of time‐to‐first‐event of incident VA: hazard ratio (HR): 1.39, 95% confidence interval (CI): 1.22–1.58 per log unit increase, *p* < .001 (Table 2). This association persisted after adjusting for age, sex, and BMI (HR: 1.37 [95% CI: 1.20–1.58], *p* < .001), and after additionally adjusting for CAD, HF, eGFR, and LVEF (HR: 1.22 [95% CI: 1.03–1.45], *p* = .02). Patients in the highest quartile of NT‐proBNP had almost fourfold higher risk of VA compared with the lowest quartile (HR: 3.86 [95% CI: 2.10–7.10], *p* < .001) (Figure 1). The C‐statistics for NT‐proBNP in predicting VA was 0.62 [95% CI: 0.57–0.67].

**Table 2:** Association between NT‐proBNP and the risk of VA, hospitalization for HF, and all‐cause mortality.

	C‐statistics	Cox regression – Unadjusted model		Cox regression – Multivariable model 1^a^		Cox regression – Multivariable model 2^b^	
	Harrell's C (95% CI)	HR (95% CI)	*p*	HR (95% CI)	*p*	HR (95% CI)	*p*
VA (*n* = 137)	0.62 (0.57–0.67)	1.39 (1.22–1.58)	<.001	1.37 (1.20–1.58)	<.001	1.22 (1.03–1.45)	.02
HF hospitalization (*n* = 87)	0.85 (0.81–0.89)	3.11 (2.53–3.82)	<.001	3.38 (2.69–4.24)	<.001	3.04 (2.33–3.97)	<.001
All‐cause mortality (*n* = 76)	0.82 (0.77–0.87)	2.49 (2.04–3.03)	<.001	2.33 (1.86–2.92)	<.001	1.96 (1.50–2.58)	<.001

*Note*: Analyzed by proportional Cox regression per log unit increase of NT‐proBNP in association to events in unadjusted model and after adjustments for risk factors in two separate models. Also presented is Harrell's C‐statistics for the unadjusted model.

Abbreviations: BMI, body mass index; CAD, coronary artery disease; CI, confidence interval; GFR, glomerular filtration rate; HF, heart failure; HR, hazard ratio; LVEF, left ventricular ejection fraction; NT‐proBNP, N‐terminal pro‐B‐type natriuretic peptide; VA, ventricular arrhythmia.

^a^ Adjusted for age, sex, and BMI.

^b^ Adjusted for age, sex, BMI, CAD, HF, estimated GFR, and LVEF.

### 3.4 NT‐proBNP and risk of VA in primary and secondary prevention ICD indication

Patients with a primary prevention ICD indication had higher NT‐proBNP concentrations than patients with secondary prevention indication: median 761 (235–1818) ng/L versus 442 (192–1058) ng/L, *p* < .001 (Supporting Information: Table 1). There was a trend for a stronger association between NT‐proBNP concentrations and incident VA in patients with a secondary prevention ICD indication (HR 1.59 [95% CI 1.34–1.88], *p* < .001) compared with patients with a primary prevention ICD indication (HR 1.24 [1.02–1.51], *p* = .03), *p* = .06 (Table 3 and Figure 1). In patients with a secondary prevention indication, the association between NT‐proBNP and VA persisted in the fully adjusted model (HR 1.32 [1.02–1.70], *p* = .04), whereas it was attenuated and nonsignificant in primary prevention patients (HR 1.10 [0.87–1.40, *p* = .46). The C‐statistics for patients with secondary prevention was 0.71 (95% CI: 0.64–0.77) and primary prevention 0.55 (95% CI: 0.47–0.63).

**Table 3:** Association between NT‐proBNP and the risk of VA, hospitalization for HF, and all‐cause mortality in patients with a primary prevention ICD indication and a secondary prevention ICD indication.

	C‐statistics	Cox regression – Unadjusted model		Cox regression – Multivariable model 1^a^		Cox regression – Multivariable model 2^b^	
	Harrell's C (95% CI)	HR (95% CI)	*p*	HR (95% CI)	*p*	HR (95% CI)	*p*
Primary prevention ICD indication							
VA (*n* = 60)	0.55 (0.47–0.63)	1.24 (1.02–1.51)	.03	1.24 (1.01–1.52)	.04	1.10 (0.86–1.40)	.46
HF Hospitalization (*n* = 54)	0.81 (0.75–0.87)	3.07 (2.27–4.14)	<.001	3.25 (2.39–4.43)	<.001	3.23 (2.28–4.57)	<.001
All‐cause mortality (*n* = 46)	0.81 (0.74–0.87)	2.51 (1.91–3.30)	<.001	2.61 (1.87–3.64)	<.001	2.12 (1.46–3.07)	<.001
Secondary prevention ICD indication							
VA (*n* = 77)	0.71 (0.64–0.77)	1.59 (1.34–1.88)	<.001	1.53 (1.27–1.85)	<.001	1.32 (1.02–1.70)	.04
HF hospitalization (*n* = 33)	0.88 (0.83–0.94)	3.12 (2.33–4.18)	<.001	3.56 (2.45–5.18)	<.001	3.49 (2.03–5.99)	<.001
All‐cause mortality (*n* = 30)	0.83 (0.75–0.90)	2.42 (1.80–3.26)	<.001	2.09 (1.46–3.01)	<.001	1.90 (1.16–3.10)	.01

*Note*: Analyzed by proportional Cox regression per log unit increase of NT‐proBNP in association to events in unadjusted model and after adjustments for risk factors in two separate models. Also presented is Harrell's C‐statistics for the unadjusted model.

Abbreviations: BMI, body mass index; CAD, coronary artery disease; GFR, glomerular filtration rate; HF, heart failure; LVEF, left ventricular ejection fraction; VA, ventricular arrhythmia.

^a^ Adjusted for age, sex and BMI.

^b^ Adjusted for age, sex, BMI, CAD, HF, estimated GFR, and LVEF.

### 3.5 NT‐proBNP and associations with death and HF hospitalization

**Figure 2:**
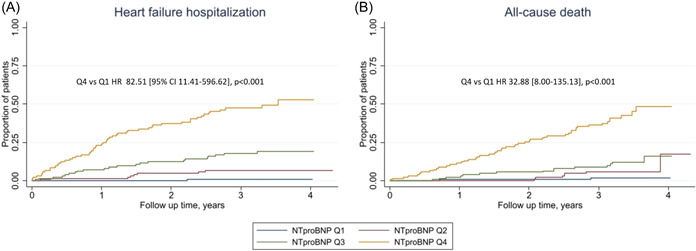
Association between baseline concentrations of N‐terminal‐pro B‐type natriuretic peptide (NT‐proBNP) and time to (A) heart failure hospitalization and (B) all‐cause death in the total population. Stratified by quartiles of NT‐proBNP and *p* is for Quartile 4 versus Quartile 1.

During follow‐up, 87 patients (18%) experienced at least 1 hospitalization for HF and 76 (16%) patients died during follow‐up, including 35 classified as cardiovascular death. Greater concentrations of NT‐proBNP were associated with a higher risk of HF hospitalization (HR 3.11 [2.53–3.82], *p* < .001; C‐statistics 0.85) (Table 2 and Figure 2A) and this persisted in adjusted models. NT‐proBNP concentrations were associated with all‐cause mortality (HR 2.49 [95% CI 2.04–3.03], *p* < .001; C‐statistics 0.82) (Table 2 and Figure 2B), which persisted in adjusted models.

### 3.6 Change in NT‐proBNP measurements from baseline to follow‐up

In total, 459 (94%) patients attended the follow‐up visit. Among the 30 nonattending patients, 25 were dead. Blood samples were collected in 411 (84%) patients, mean 1.4 ± 0.5 years after the baseline visit. Baseline characteristics of patients with and without follow‐up NT‐proBNP measurements are presented in Supporting Information: Table 3. The median NT‐proBNP concentration at the follow‐up visit was 469 (171–1202) ng/L, which was not significantly different from the baseline concentrations (*p* = .31). The relative change in NT‐proBNP from baseline to follow‐up was median −2% (−35% to 36%). Patients with greater increases in NT‐proBNP between the visits had higher baseline blood pressure, higher baseline LVEF and more frequently a secondary indication for ICD (Supporting Information: Table 4). Changes in NT‐proBNP were not associated with subsequent incident VA (*N* = 46; HR: 1.00 [95% CI: 0.66–1.52] *p* = .98). Greater changes in NT‐proBNP associated with an increased risk of subsequent hospitalization for HF (*N* = 34; HR: 1.73 [95% CI: 1.03–2.90], *p* = .04 and all‐cause death (*N* = 42; HR: 1.71 [95% CI: 1.05–2.77], *p* = .03) in the fully adjusted model. These results were consistent when analyzing absolute changes in NT‐proBNP.

## 4 DISCUSSION

We report the following main findings: (1) Higher concentrations of NT‐proBNP predict the risk of incident VA, with an almost fourfold increased risk in patients with NT‐proBNP in the highest quartile (>~1500 ng/L) compared with the lowest quartile (<~200 ng/L). (2) This association was independent of established risk factors for cardiac arrest such as age, sex, CAD, renal function, and most importantly, LVEF. (3) The association appeared to be stronger in patients with secondary prevention than primary prevention ICD indication. (4) There was no association between change in NT‐proBNP levels over ~1.5 years and the risk of subsequent VA.

### 4.1 NT‐proBNP as a predictor for major cardiovascular events

Elevated levels of circulating NT‐proBNP are common in patients with HF and measurements are recommended for diagnostic and prognostic purposes.^6^, ^13^, ^14^, ^15^, ^16^ The association between NT‐proBNP levels and cardiovascular risk has also been demonstrated in lower risk cohorts, including community‐based studies of individuals free of HF.^8^, ^17^, ^18^ In our study we extend these findings to patients treated with ICD at very high cardiovascular risk by showing a strong association between higher baseline NT‐proBNP concentrations and an increased risk of VA, HF‐hospitalization, and all‐cause death. NT‐proBNP performed better at predicting the risk of HF hospitalization and mortality compared with VA risk. This finding is in line with previous studies suggesting NT‐proBNP to be a strong prognostic marker of worsening HF status and all‐cause death due to the range of pathophysiology (i.e., aging, renal function, and myocardial stress) reflected by elevated levels.

### 4.2 NT‐proBNP as a predictor for VAs and SCD

Although no specific mechanisms have linked NT‐proBNP directly with risk of VA and SCD, myocardial stretch, which is the main stimulus for the synthesis and secretion of natriuretic peptides,^19^ have been proposed as a potential arrhythmic trigger.^7^, ^11^, ^20^ Myocardial stretch can trigger mechano–electrical feedback leading to complex electrophysiological disturbances that can enhance different arrhythmogenic processes, triggering automaticity, triggered activity and reentry.^21^, ^22^, ^23^ Previous studies have suggested that NT‐proBNP can help predict SCD,^6^, ^7^, ^8^, ^9^ however, with different definitions of SCD. The majority of studies in this field define SCD as sudden and unexpected death, presumed to be arrhythmic occurring within 1 h of onset of symptoms, or if the deceased has been witnessed to be stable within 24 h of the arrest in case of unwitnessed death.^6^, ^8^, ^10^, ^24^ Diverging and vague definitions of SCD are unfortunate limitations of many of the published studies as it does not rule out other nonarrhythmic sudden death etiologies. An important strength of our study is that we included patients with implanted ICD with the advantage of having documented arrhythmias with relatively long follow‐up. We also analyzed NT‐proBNP in one batch from blood samples stored in a dedicated biobank, which reduces the risk of analytical bias. In concordance with other studies of patients with ICD our results demonstrate a significant association between NT‐proBNP and incident VA.^25^, ^26^, ^27^

In a meta‐analysis, LVEF was demonstrated to not influence the association between natriuretic peptides and SCD in patients with and without ICD.^7^ Our findings support that the association between elevated NT‐proBNP and VA is independent of LVEF.

### 4.3 NT‐proBNP in primary versus secondary prevention

Our results suggest that there was an interaction by ICD indication on the association between NT‐proBNP and risk of VA. The ability to discriminate between patients with and without incident VA was stronger in patients with a secondary ICD‐indication, and for these patients the association persisted in adjusted models. In patients with a secondary prevention ICD indication, 11% in the lowest quartile of NT‐proBNP had incident VA, whereas 52% had incident VA in the highest quartile. Guidelines recommend ICD implantation in patients who have experienced VA with hemodynamic consequences or within 48 h after myocardial infarction, in the absence of reversible causes. ^28^ However, there may be uncertainties related to whether the cause of VA is reversible and many patients with a low‐risk of recurrent events never experience subsequent events. In these settings, our data support that NT‐proBNP measurement may be helpful in assessing the risk of future VA, although this should be validated in future prospective cohorts.

Among patients with a primary ICD indication in our study, the performance of NT‐proBNP in predicting VA was limited. Potential explanation for this may be that patients with advanced HF have non‐arrhythmic mechanisms driving NT‐proBNP secretions, such as neurohormonal activation and renal dysfunction.^29^, ^30^ This is supported by our finding of a strong association between NT‐proBNP and the risk of all‐cause death and HF hospitalization in these patients. Thus, our findings suggest that it is challenging to use NT‐proBNP as a marker specifically for VA risk in patients considered for primary prevention ICD.

### 4.4 Change in NT‐proBNP from baseline to follow‐up visit

There were no significant changes in NT‐proBNP concentrations from baseline to the follow‐up visit. Moreover, we found no association between the change in NT‐proBNP concentration and the risk of subsequent incident VA. Although serial measurements of NT‐proBNP may be useful in assessing HF status, our findings argue against repeated measurements for the purpose of arrhythmic risk stratification.

#### 4.4.1 Study limitations

Our cohort consisted of patients treated with ICD with high arrhythmic risk, and whether our results are applicable to other patient population is uncertain. The majority of patients in our study were men, which also is the case in similar cohorts. Women have intrinsically higher levels of NT‐proBNP than men, and whether the results can be generalized to women is less certain. However, in the Nurses’ Health Study, NT‐proBNP was associated with the risk of SCD in 121 700 women.^24^ Analytical variability of NT‐proBNP measurements may be a reason for bias, which we tried to overcome by analyzing all samples in one batch using the same assay and instruments. Survival bias may have been introduced for the analysis using serial sampling, as death was the most important reason for nonattendance at the follow‐up visit. The analysis stratified for ICD indication was posthoc and with limited power and must therefore be considered hypothesis‐generating.

## 5 CONCLUSIONS

In our cohort of patients with ICD, we found a significant association between high NT‐proBNP concentrations and the risk of developing VA, as well as HF hospitalization and death, independent of established risk factors. NT‐proBNP is a noninvasive test that is widely available and reproducible. Our data suggest that NT‐proBNP may be a helpful tool for assessing VA risk, particularly in patients with a secondary ICD indication.

## CONFLICT OF INTEREST STATEMENT

H. Røsjø has received personal fees from Novartis and Thermo Fischer BRAMHS, CardiNor, and SpinChip Diagnostics. T. Omland has served on advisory boards for Abbott Diagnostics, Roche Diagnostics, and Bayer, and has received research support from Abbott Diagnostics, Novartis, Roche Diagnostics, Singulex, and SomaLogic via Akershus University Hospital, and speaker's or consulting honoraria from Roche Diagnostics, Siemens Healthineers and CardiNor. P. L. Myhre has served on advisory boards and received speaker fees from Amarin, AmGen, AstraZeneca, Bayer, Boehringer Ingelheim, Novartis, and Novo Nordisk. All other authors report no conflict of interest.

## Supporting information

Supporting Information.

## Data Availability

Research data are not shared.
