# Supplementary material for: N‐terminal pro‐B‐type natriuretic peptide for prediction of ventricular arrhythmias: Data from the SMASH study
Source: Clin Cardiol. 2023 Jul 3;46(8):989–96. doi: 10.1002/clc.24074 (PMC10436794; doi:10.1002/clc.24074)
Supplement: Supplementary file 1 — Supporting Information. [file CLC-46-989-s001.docx]

**Supplementary Material**

**to**

**N-terminal pro-B-type natriuretic peptide for prediction of ventricular arrhythmias; data from the SMASH Study**

**Supplemental Table 1** Baseline characteristics of the study population according to the ICD indication

|  | Primary prevention | | Secondary prevention  (n=240) | P-value for primary vs secondary |
| --- | --- | --- | --- | --- |
|  | **HF indication**  **(n= 225)** | **Other indications**  **(n=25)** |  |  |
| **Age, y** | 67.6 ± 10.8 | 57.2 ± 16.6 | 65.7 ± 12.9 | 0.45 |
| **Male sex** | 194 (86.6%) | 18 (72.0%) | 195 (81.2%) | 0.25 |
| **Body mass index, kg/m2** | 27.8 ± 5.4 | 27.0 ± 3.3 | 27.6 ± 4.2 | 0.77 |
| **Systolic blood pressure, mmHg** | 121 ± 20 | 127 ± 18 | 129 ± 20 | <0.001 |
| **Diabetes mellitus** | 50 (22.2%) | 2 (8.0 %) | 42 (17.5%) | 0.35 |
| **Coronary artery disease** | 146 (65.5%) | 6 (24.0%) | 157 (66.8%) | 0.21 |
| **Previous acute myocardial infarction** | 126 (56.8%) | 2 (8.0 %) | 148 (61.7%) | 0.03 |
| **Cardiomyopathy** | 4 (1.8 %) | 17 (68.0%) | 13 (5.4 %) | 0.19 |
| **Heart failure** | 225 (100.0%) | 0 (0.0%) | 168 (70.4%) | <0.001 |
| **Left ventricular ejection fraction, %** | 35 ± 11 | 57 ± 6 | 44 ± 12 | <0.001 |
| **New York Heart Association class III-IV** | 29 (12.9%) | 1 (4.0 %) | 22 (9.2 %) | 0.31 |
| **Previous documentation of ventricular arrhythmia** | 57 (25.6%) | 7 (28.0%) | 218 (90.8%) | <0.001 |
| **Estimated glomerular filtration rate, ml/min/1.73 m^2^** | 69 ± 24 | 97 ± 28 | 75 ± 23 | 0.14 |
| **NT-proBNP, ng/L** | 831 (288-1920) | 169 (116-516) | 442 (192-1058) | <0.001 |

**Supplemental Table 2.** Predictors of baseline NT-proBNP levels assessed by multivariable logistic regression models including all variables in the table.

|  | Beta coefficient  [95% CI] | Z-value | P-value |
| --- | --- | --- | --- |
| Age | 0.01 [0.004-0.02] | 2.8 | 0.005 |
| Male sex | -0.22 [-0.50-0.05] | -1.6 | 0.11 |
| Body mass index, kg/m2 | -0.45 [-0.67- -0.24] | -4.2 | <0.001 |
| Systolic blood pressure, mmHg | -0.001 [-0.051- -0.053] | 0.03 | 0.98 |
| Diabetes mellitus | 0.26 [-0.01-0.51] | 2.0 | 0.05 |
| Previous acute myocardial infarction | 0.17 [-0.05-0.40] | 1.4 | 0.17 |
| Heart failure | 0.49 [0.16-0.82] | 3.0 | 0.003 |
| Left ventricular ejection fraction, % | -0.03 [-0.04- -0.02] | -6.7 | <0.001 |
| New York Heart Association class III-IV | 0.37 [0.06-0.70] | 2.3 | 0.02 |
| Cardiomyopathy | 0.71 [0.24-1.17] | 3.0 | 0.003 |
| Previous documentation of ventricular arrhythmias | 0.05 [-0.15-0.26] | 0.6 | 0.54 |
| Estimated glomerular filtration rate, ml/min/1.73 m^2^ | -0.01[-0.02—0.01] | -5.6 | <0.001 |

**Suppl. Table 3** Baseline characteristics of the study population in patients with available blood samples at the follow-up visit and those without.

|  | Available blood samples at the follow-up visit    *n=411* | Not available blood samples at the follow-up visit  *n=79* | P-value |
| --- | --- | --- | --- |
| Age, y | 65.9 ± 12.2 | 67.1 ± 13.3 | 0.45 |
| Male sex | 339 (82.5%) | 69 (87.2%) | 0.31 |
| Body mass index, kg/m2 | 28.0 ± 4.8 | 26.1 ± 4.1 | 0.002 |
| Systolic blood pressure, mmHg | 125 ± 20 | 126 ± 23 | 0.63 |
| Diabetes mellitus | 72 (17.5%) | 22 (27.8%) | 0.03 |
| Coronary artery disease | 259 (64.0%) | 50 (64.1%) | 0.98 |
| Previous acute myocardial infarction | 227 (55.6%) | 49 (62.0%) | 0.29 |
| Heart failure | 324 (78.8%) | 70 (88.6%) | 0.05 |
| Left ventricular ejection fraction, % | 41 ± 13 | 38 ± 12 | 0.03 |
| New York Heart Association class III-IV | 37 (9.0 %) | 15 (19.0%) | 0.01 |
| Cardiomyopathy | 29 (7.1 %) | 5 (6.4 %) | 0.84 |
| Previous documentation of ventricular arrhythmias | 240 58.5%) | 42 (53.8%) | 0.44 |
| Primary ICD indication | 204 (49.6%) | 46 (58.2%) | 0.16 |
| Estimated glomerular filtration rate, ml/min/1.73 m^2^ | 74.6 ± 22.7 | 68.4 ± 29.7 | 0.04 |
| Baseline NT-proBNP, ng/L | 511 (178-1313) | 1248 (524-2858) | <0.001 |

**Suppl. Table 4** Baseline characteristics by quartiles of relative change in NT-proBNP from baseline to the follow-up visit

|  | NTproBNP  Change Q1  n=103 | NTproBNP Change Q2  n=103 | NTproBNP Change Q3  n=103 | NTproBNP Change Q4  n=102 | P-value for trend |
| --- | --- | --- | --- | --- | --- |
| *Relative change in NT-proBNP (median Q1-Q3)* | *-50% (-60% to -43%)* | *-18% (-26% to -10%)* | *12% (6% to 24%)* | *84% (60% to 141%)* |  |
| Age, y | 65.1 ± 13.0 | 65.0 ± 12.0 | 66.5 ± 11.6 | 67.2 ± 12.2 | 0.14 |
| Male sex | 81 (78.6%) | 85 (82.5%) | 85 (82.5%) | 88 (86.3%) | 0.17 |
| Body mass index, kg/m2 | 28.8 ± 5.5 | 27.8 ± 4.3 | 27.7 ± 4.6 | 27.6 ± 4.5 | 0.07 |
| Systolic blood pressure, mmHg | 122 ± 20 | 124 ± 16 | 126 ± 21 | 127 ± 21 | 0.046 |
| Diabetes mellitus | 21 (20.4%) | 13 (12.6%) | 16 (15.5%) | 22 (21.6%) | 0.71 |
| Coronary artery disease | 60 (59.4%) | 66 (64.7%) | 74 (72.5%) | 59 (59.0%) | 0.75 |
| Previous acute myocardial infarction | 53 (52.0%) | 57 (55.3%) | 65 (64.4%) | 52 (51.0%) | 0.79 |
| History of heart failure | 84 (81.6%) | 81 (78.6%) | 84 (81.6%) | 75 (73.5%) | 0.24 |
| Left ventricular ejection fraction, % | 39 ± 12 | 40 ± 14 | 40 ± 13 | 44 ± 12 | 0.005 |
| New York Heart Association class III-IV | 6 (5.8 %) | 8 (7.8 %) | 15 (14.6%) | 8 (7.8 %) | 0.31 |
| Cardiomyopathy | 7 (6.8 %) | 10 (9.7 %) | 7 (6.8 %) | 5 (4.9 %) | 0.45 |
| Previous documentation of ventricular arrhythmias | 58 (56.3%) | 57 (55.3%) | 60 (58.3%) | 65 (64.4%) | 0.22 |
| Primary ICD indication | 58 (56.3%) | 53 (51.5%) | 50 (48.5%) | 43 (42.2%) | 0.04 |
| Estimated glomerular filtration rate, ml/min/1.73 m^2^ | 74.6 ± 22.5 | 74.4 ± 21.9 | 77.9 ± 24.6 | 71.5 ± 21.6 | 0.58 |


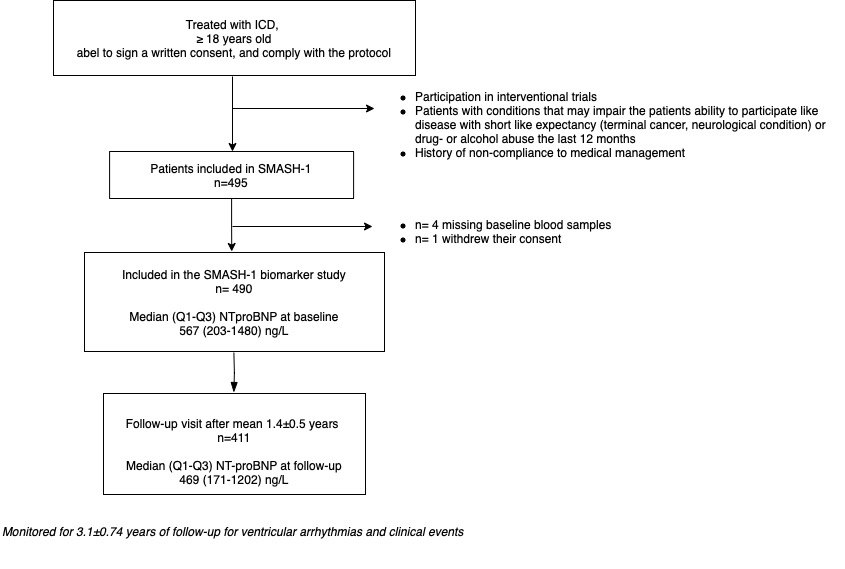
**Supplemental Figure 1.** Flow diagram of the SMASH 1 biomarker study
